# Equine sarcoids: Bovine Papillomavirus type 1 transformed fibroblasts are sensitive to cisplatin and UVB induced apoptosis and show aberrant expression of p53

**DOI:** 10.1186/1297-9716-43-81

**Published:** 2012-12-04

**Authors:** Margaret Finlay, ZhengQiang Yuan, Iain M Morgan, M Saveria Campo, Lubna Nasir

**Affiliations:** 1 MRC-University of Glasgow Centre for Virus Research, Institute of Infection, Inflammation and Immunity, College of Medical, Veterinary and Life Sciences, University of Glasgow, Bearsden Road, Glasgow, Scotland, G61 1QH, United Kingdom; 2 Division of Life Sciences, University College, London, 5 University Street, London, WC1E 6JF, United Kingdom

## Abstract

Bovine papillomavirus type 1 infects not only cattle but also equids and is a causative factor in the pathogenesis of commonly occurring equine sarcoid tumours. Whilst treatment of sarcoids is notoriously difficult, cisplatin has been shown to be one of the most effective treatment strategies for sarcoids. In this study we show that in equine fibroblasts, BPV-1 sensitises cells to cisplatin-induced and UVB-induced apoptosis, a known cofactor for papillomavirus associated disease, however BPV-1 transformed fibroblasts show increased clonogenic survival, which may potentially limit the therapeutic effects of repeated cisplatin treatment. Furthermore we show that BPV-1 increases p53 expression in sarcoid cell lines and p53 expression can be either nuclear or cytoplasmic. The mechanism and clinical significance of increase/abnormal p53 expression remains to be established.

## Introduction

Sarcoids are the most common skin tumours to affect equids worldwide [1-3] with reported prevalence rates ranging from 0.5% to 2.0% [4,5]. Recently, high prevalence rates (25-53%) have been reported in two populations of inbred zebras [6]. Equine sarcoid affects horses of all ages, breeds and colour with no sex predilection. Six clinical types of sarcoids are recognised including occult, verrucose, nodular, fibroblastic, mixed and malignant types [7].

Treatment of sarcoid is notoriously difficult and currently there is no effective treatment for sarcoids that cures without recurrence [7-9]. Surgery is often used but has a high failure rate due to tumour recurrence [8]. As a result of the difficultly in treating sarcoids, there are a variety of treatment options that have been used in clinical practice including cryosurgery [10] laser surgery [11], BCG immunotherapy [12], intratumoral chemotherapy and topical formulations including zinc chloride cream [13] imiquimod [14] or aciclovir [15]. The success of therapy appears to depend on several factors including site and size of the tumour, type of sarcoid and number of lesions [16,17]. Furthermore, it is generally accepted that prognosis for treatment is worse if one or more unsuccessful treatment attempts have previously been made [16].

It is now well established that equine sarcoids are caused by infection with Bovine Papillomaviruses (BPV) types 1 and 2 [18]. BPV-1/2 are non-enveloped double stranded DNA viruses with a genome of approximately 8kbp that infect their natural host, cattle, causing papillomas of skin or mucosa which generally regress without eliciting any serious clinical problems in the host [19]. None of the treatment strategies for sarcoids directly target the viral infection, although vaccine studies in vivo have been successful [20] and in vitro studies to knock down viral DNA expression are very effective in preventing growth of sarcoid fibroblasts [21,22]. Recently we have developed a panel of BPV-1 transformed equine cell lines as model systems for studying the pathogenesis of equine sarcoids [23], and these lines have proved invaluable in our understanding of sarcoid disease pathogenesis [21-28]. Using these cell lines, in the present study we have evaluated the cell killing efficacy of the chemotherapeutic agents cisplatin and the effects of Ultraviolet B (UVB). Cisplatin has been shown to be one of the most effective treatment strategies for sarcoids with success rates close to 100% [29-31]. Cisplatin causes cross-links in DNA, leading to DNA functional abnormalities culminating in the initiation of cell death via caspases [32] by activation of mitogen-activated protein kinase (MAPK) signaling pathway [33]. UV is a known cofactor for PV associated disease [34] and whilst there is no evidence that UVB is involved in equine sarcoid pathogenesis, previous studies have shown that papillomaviral proteins can inhibit UV induce apoptosis [35].

## Materials and methods

### Cell culture

Normal equine embryonic fibroblast line EqPalF, BPV-1 in vitro transformed EqPalF (S6-1, S6-2, S6-3), sarcoid cell lines EqS01a, EqS02a, EqS03a and EqS04b have differing viral loads and viral gene expression levels and have been described previously [23]. EqS03a and EqS013 are new sarcoid derived cell lines generated as described previously [23]. Cells were maintained in culture in complete DMEM in a 37°C humidified atmosphere of 5% CO_2_ in air.

### Sarcoid tissue samples

Sarcoid tumour samples were collected with informed owner consent from cases presented at the Weipers Centre for Equine Welfare, University of Glasgow and at the Liverpool University Equine Hospital.

### Apoptosis

To assess apoptosis, cells were double-stained with FITC-Annexin V and propidium iodide (PI) using the Annexin V Apoptosis Detection kit (Darmstadt, Germany), according to the manufacturer’s recommendations. Cells were assessed 24 h post UVB treatment (Ultaviolet transilluminator, Sigma, Dorset, UK; 250 and 500 mJ/cm^2^). Cells were incubated with cisplatin (10 μg/mL and 25 μg/mL, Hospira, Warickshire, UK) for 72 h and then assessed for apoptosis. All cells were analyzed on an Epics XL Flow Sorter (Beckman-Coulter, High Wycombe, UK). For analysis, the cells were divided into four distinct populations using the control cells as a reference: costaining with Annexin V and PI allows differentiation of viable non apoptotic cells (Annexin V^−^, PI^−^) from early apoptotic cells (Annexin V^+^, PI^−^) and late apoptotic cells (Annexin V^+^, PI^+^). The cells in each quadrant were gated, and the percentage of the total cell population was determined.

### Clonogenic survival assays

Following exposure of cells to either UVB or cisplatin (described above), approx 5 × 10^3^ cells were washed and suspended in fresh medium and then serial dilutions were plated onto methylcellulose-containing medium. Ten days later, the cells were fixed and stained with 10% methylene blue in 70% ethanol. The number of colonies were counted, and the surviving fraction was calculated as the ratio of the number of colonies in the treated sample to the number of colonies in the untreated sample. All tests were performed in triplicate.

### P53 expression

To assess endogenous and DNA damage-induced stabilization of p53, cells were plated at 8 × 10^5^ in 10 cm^2^ petri dishes and were irradiated with UV light at 250 J/m^2^ and harvested 4 h post UV treatment. Cells were lysed by the addition of 500 μL of 2 × SDS sample buffer. Samples were boiled, analyzed by SDS 10% PAGE, and assessed by Western blotting using human p53 D0-7 antibody (DO-7 p53 clone; Novocastra Milton Keynes, UK diluted 1:200 in 10 mM Tris Buffer Saline (TBS) using standard methods. Filters were stripped and reprobed with GAPDH antibody (V-18 Novacastra) to control for protein loading. To assess the transcriptional activation function of p53, cells were irradiated with UV light at 30 J/m^2^ and harvested ~10 h post UV treatment for assessment of p21 expression (C-19, Novacastra). The half-life of the p53 protein was determined following culturing of cells with or without cyclohexamide (40 μg/mL, 48 h) at 37°C. Cells were harvested at various time points (0, 0.5, 1, 3, 5 h) and assessed for p53 protein expression by western blot analysis using D0-7 antibody. Quantification of expression levels was carried out by using NIH ImageJ Software analysis (NIH, Maryland, USA).

The assessment of p53 protein expression in sarcoid tissue sections or cells grown on 8-well chamber slides was performed using a two-step immunohistochemical technique using Dako EnVision kit (K4007, Cambridgeshire, UK) following the manufacturer’s instructions using D0-7 p53 antibody (1:400). For negative controls, duplicate slides were incubated with a non-related serum instead of the p53 primary antibody.

## Results

### BPV-1 confers increased sensitivity to UVB and cisplatin induced apoptosis

**Figure 1:**
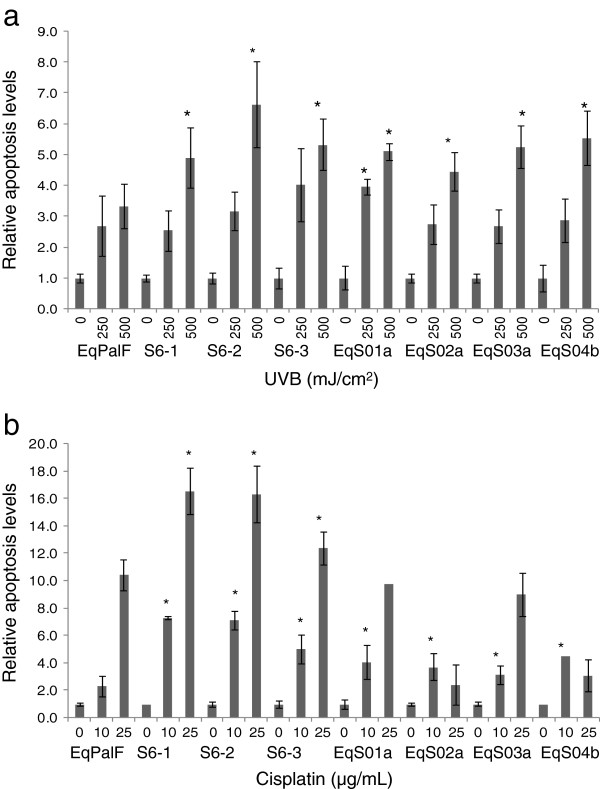
**Cisplatin and UVB induced apoptosis.** Levels of apoptosis detected in control (EqPalF), BPV-1 transformed EqPalFs (S6 cells) and sarcoid cell lines (EqS cells) following treatment with (**a**) UVB (**b**) cisplatin. Results are shown relative to untreated cells. Experiments were performed in triplicate. *indicates significance (*p* < 0.05).

To determine whether expression of BPV-1 can influence apoptosis, BPV-1 transformed EqPalF cells (S6 cells), control EqPalF cells and a panel of sarcoid derived cell lines (EqS) were exposed to UVB and cisplatin followed by quantitative assessment by FACs analysis. Firstly, cells were exposed to UVB irradiation (250, 500 mJ/cm^2^) and apoptosis assessed 24 h post irradiation (Figure 1a). An increase in cell apoptosis was detected in S6 cells compared to the parental cell line which lacks BPV-1 (EqPalF), indicating that BPV-1 confers sensitivity to UVB induced apoptosis. An increase in cell apoptosis was also detected in sarcoid derived tumour lines (Figure 1a). The same panel of cell lines were treated with cisplatin (10, 25 μg/mL) for 72 h. A significant increase in apoptosis was observed in S6 cells (Figure 1b) demonstrating that BPV-1 sensitises cells to cisplatin induced apoptosis. Increased apoptosis was also detected in sarcoid derived tumour lines in the presence of 10 μg/mL cisplatin but not at a higher dose (Figure 1b). The sensitivity to UVB and cisplatin induced apoptosis is independent of viral load or viral gene expression.

### BPV-1 increases clonogenic survival following exposure to DNA damaging agents

**Figure 2:**
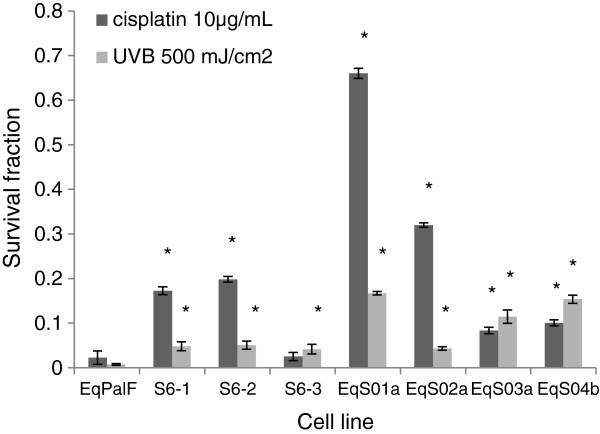
**Clongenic survival following cisplatin and UVB exposure.** Clonogenic survival of control (EqPaLF), BPV-1 transformed EqPalFs (S6 cells) and sarcoid cell lines (EqS cells) following treatment with cisplatin and UVB. The surviving fraction is the ratio of the number of colonies in the treated sample to the number of colonies in the untreated sample. All tests were performed in triplicate. *indicates significance (*p* < 0.05).

To determine the sensitivity/resistance of BPV transformed cells to the cytotoxic effects of DNA damaging agents following exposure to DNA damaging agents, cells were subjected to clonogenic cellular survival assays. The survival fractions following irradiation with 500 mJ/cm^2^ UVB of all S6 and sarcoid cell lines showed increased clonogenic survival than control cells (Figure 2). Similarly following cisplatin (10 μg/mL) treatment, the majority of cell lines showed increased cell survival (Figure 2).

### BPV-1 increases p53 expression

**Figure 3:**
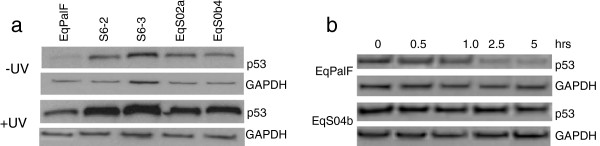
**P53 expression. a**. UVB induced p53 expression in control (EqPalF), BPV-1 transformed EqPalFs (S6 cells) and sarcoid cell lines (EqS cells). **b**. p53 protein half-life in control EqPalF and EqS04b cells.

Since UVB and cisplatin can induce apoptosis via p53 dependent pathways, we next sought to address the expression levels of p53 in the cell lines. p53 protein expression was compared by Western blot analysis using D0-7 antibody in the control cell line EqPalF, two BPV-1 transformed cell lines (S6-2 and S6-3) and two sarcoid derived cell lines (EqS02a and EqS04b). As shown in Figure 3a, all BPV-1 cell lines examined showed higher levels of p53 protein expression compared to control EqPalF cells (which express negligible/very low levels of p53) and UVB exposure further increased p53 expression. These data demonstrate that p53 expression is increased by BPV-1 and that UVB increases p53 (Figure 3a).

To establish whether the increased levels of p53 expression in BPV-1 fibroblasts compared to EqPalFs was due to increased stability, EqS04b cells were treated with cyclohexamide to block protein synthesis and p53 expression examined at various time points. As shown in Figure 3b, p53 expression remained unchanged in EqS04b cells whereas it diminishes over time in control cells. This suggests that p53 upregulation in BPV-1 cells is due to enhanced p53 stability.

**Figure 4:**
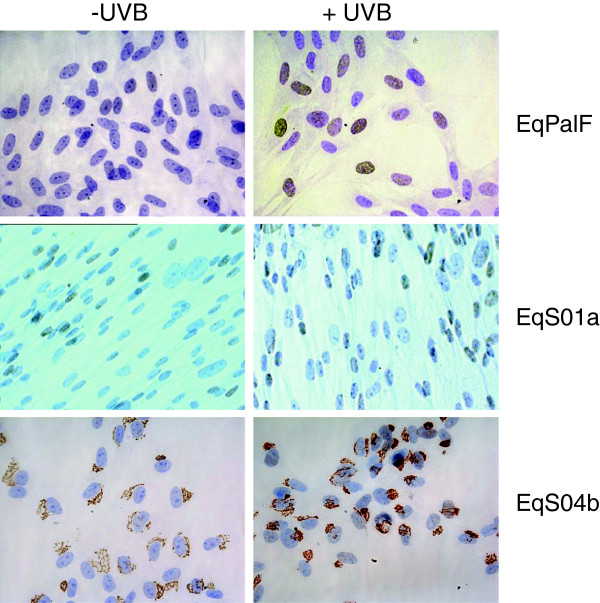
**Immunohistochemical detection of p53.** P53 expression prior to and following UVB exposure.

**Figure 5:**
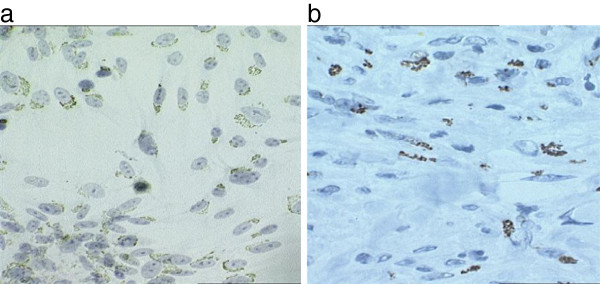
**Cytoplasmic expression of p53.** Cytoplasmic/perinuclear p53 staining in EqS013 cells and the tumour from which the cell line was derived.

To further investigate the expression pattern of p53, Immunocytochemistry (ICC) was performed prior to and following UVB irradiation in two sarcoid derived cell lines (EqS01a, EqS04b) and the BPV-1 negative line. EqPalFs did not express any detectable p53 by ICC, however following UVB exposure, p53 protein expression was induced in the nuclei of cells (Figure 4). EqS01a cells showed detectable nuclear p53 expression in the absence of UVB and the percentage of positive cells increased following UVB exposure (Figure 4). These results are in agreement with the western blot data. In contrast, EqS04b cells showed a distinct punctate cytoplasmic pattern with no nuclear staining evidence (Figure 4). The levels of cytoplasmic p53 expression following UV irradiation also increased in these cells (Figure 4), in agreement with the Western blot data. Since this pattern of p53 expression was unexpected, we performed ICC on further cell lines and a similar staining pattern was observed for cell line EqS013 (Figure 5a). The original tumour sample from which the cell line EqS013 was derived was also examined for p53 expression and cytoplasmic staining was clearly evident in the tumour biopsy (Figure 5b) indicating that the aberrant p53 expression is not a result of cell culture.

### P53 expression in vivo

To establish whether there was any association between levels of p53 expression and the clinical type of sarcoid, 39 sarcoid tumours were subjected to Immunohistochemistry (IHC) using the D0-7 antibody. The results are presented in Table 1. Of the 39 sarcoids tested, 17 (43%) were positive for nuclear p53 expression and most p53 positive tumours were fibroblastic or nodular tumour types.

**Table 1:** Levels of p53 immunoreactivity in different clinical sarcoid types

**p53 expression**	**Fibroblastic**	**Nodular**	**Verrucose**	**Mixed**	**Total**
neg	3	10	3	6	22
low	1	1	0	2	4
med	3	4	0	0	7
high	5	1	0	0	6
	12	16	3	8	39

(Low = 0-10% cells positive, Med = 10-20% cells positive, High > 20% cells positive).

## Discussion

Currently, there is no universally effective therapy for the treatment of sarcoids which remains a major clinical challenge. The efficacy of different treatments is difficult to assess because most studies have not been designed to include controls and are frequently based on referral populations of horses treated at veterinary hospitals [5,36,37]. In the present study we have examined the efficacy of two DNA damaging agents to induce apoptosis.

Our data show that BPV-1 cells are more sensitive to the apoptotic effects of both UVB and cisplatin compared control cells. UVB reduces the half-life of E2 [38]; and we have shown that loss of BPV-1 E2 leads to increased apoptosis [21] which may explain this finding. The BPV-1 mediated sensitisation to cisplatin may explain the relatively good success rates and low toxicity reported with cisplatin in equine sarcoids; Theon et al., demonstrated cisplatin to be effective over a two year period in 90% of cases [29,39,40]; Hewes and Sullins [30] have reported an 85% success rate in equine sarcoids after a 2 year follow-up. Despite the increased apoptosis detected, BPV-1 cells are better able to survive the DNA insult compared to control cells. This is likely due to a small subset of resistant clones and the repeated use of cisplatin may therefore select for cells that are particularly resistant to apoptosis suggesting that cisplatin may not be appropriate for repeated treatment of sarcoids.

We have shown that p53 is overexpressed in BPV-1 cell lines. The p53 tumor suppressor protein plays a key role in coordinating cell cycle arrest, DNA repair, and programmed cell death following DNA damage [41]. P53 protein is relatively unstable and has a short half-life, and is usually undetectable in normal cells by immunohistochemistry [42]. In contrast, overexpression of p53 is often associated with the presence of mutant p53, which has a longer half life. However, there is no evidence to date for the presence of p53 gene mutations in equine sarcoids [43,44] and our sequence analysis of EqS04b cells demonstrates wild type p53 (data not shown), although the possibly of p53 gene mutations in the other cell lines cannot be ruled out. Overexpression of wild type p53 has been observed in tumours [45,46] and whilst the reason for this or its biological significance is not known, several studies have shown that it may be due to changes in the functionality of proteins that interact and control the activity and the levels of p53, such as MDM2 [47] and MDMX [48]. It has also been suggested that the overexpression may be a result of downregulation of ubiquitin mediated proteasome degradation [49]. We show that the overexpression of wild type p53 in EqS04b cells is (at least in part) due to abnormal p53 stability however further studies are necessary to understand the mechanisms involved and the clinical significance. In HPV, p53 expression is stabilised by the E7 oncoprotein [50].

We show that in 2 sarcoid derived cell lines (EqS04b, EqS013) p53 overexpression is cytoplasmic. Cytoplasmic sequestration of p53 has been proposed as an important mechanism to disrupt its function as a tumor suppressor and has been reported in a range of tumours including breast cancers and neuroblastomas [51,52]. Cytoplasmic sequestration of wild type correlates with attenuation of DNA damage-induced G1 arrest [53] and apoptosis in neuroblastoma cell lines [54]. Several factors are known to induce p53 cytoplasmic localisation including Jab1, which facilitates p53 nuclear exclusion and degradation [55] and, Parc, a parkin-like ubiquitin ligase, has recently been shown to function as cytoplasmic anchor protein of p53 [56]. The pattern of cytoplasmic staining seen in this study using the D0-7 antibody in EqS04b cells is very different from the disperse uniform cytoplasmic staining of the cytoplasm we have previously observed with CM-1 antibody [23]. In fact, in our previous study, CM-1 antibody showed cytoplasmic staining in EqS01a and S6-2 cells but not in control EqPalFs, however with the D0-7 antibody expression is clearly nuclear and expression is induced following UV exposure. These data suggest that CM-1 antibody and D0-7 antibody may recognise different forms of p53; additionally it appears that D0-7 antibody is best able to recognise equine p53 [57,58]. We and others have previously shown that a subset of equine sarcoid tumours express cytoplasmic p53 [57-60] We have also shown that in an equine sarcoid derived cell line sarc-1 with cytoplasmic p53, no mRNA expression of the p53 target gene, mdm2 could be detected either before or after UV treatment [60] showing that p53 transcriptional activation function is compromised in cells with cytoplasmic p53. The aberrant expression of p53 may also contribute to the increased clonogenic survival following DNA damage insult, but this speculative and remains to be established.

Our preliminary analysis of p53 expression and clinical type of sarcoid shows that more sarcoid aggressive sarcoids (fibroblastic and nodular) have higher levels of p53 positivity than quiescent verrucose sarcoids. However, the numbers of samples in this study are small and further studies are warranted to substantiate this initial finding.

In summary, we show that BPV-1 transformed equine fibroblasts are more sensitive to the apoptotic effects of cisplatin and UVB than control cells but show increased clonogenic survival. We show that in sarcoid cell lines, BPV-1 increases p53 expression within the nucleus but in some cell lines, p53 expression is cytoplasmic.

## Competing interests

The authors declare that they have no competing interests.

## Authors’ contributions

MF carried out all of the laboratory work in this study and assisted in the design of the study. ZQY participated in the design of the study and contributed to the drafting of the manuscript, LN participated in the design of the study, conceived the p53 study, performed the statistical analysis and coordinated the project. IM conceived the apoptosis and clonogenicity study, and participated in its design and coordination. MS assisted in the design of the p53 studies, participated in the study design and coordination and the drafting of the manuscript. All authors read and approved the final manuscript.
